# Wound dressings incorporating microRNAs: Innovative therapy for diabetic wound treatment

**DOI:** 10.22038/IJBMS.2022.67236.14739

**Published:** 2022-09

**Authors:** Mohammad Hasan Soheilifar, Nastaran Masoudi-Khoram

**Affiliations:** 1 Department of Medical Laser, Medical Laser Research Center, Yara Institute, ACECR, Tehran, Iran; 2 Department of Biophysics, Faculty of Biological Sciences, Tarbiat Modares University, Tehran, Iran

**Keywords:** Diabetic foot, Dressings, MicroRNA, Wound healing, Wound therapy

## Abstract

Diabetic wounds are the most critical complication in patients with diabetes, which often lead to hospitalization and limb amputations. Diabetic foot ulcers (DFU) is characterized by infections, prolonged inflammation, and a delayed wound healing process. Different types of medical procedures including surgical therapy, drug delivery, stem cell therapy, and wound dressings are used to manage DFU. Bioactive dressings such as hydrogels, nanofiber, and collagens are promising materials that can accelerate the healing process. The wound dressing materials can also be loaded with bioactive molecules like nucleic acids. MicroRNAs (miRNAs) are small non-coding RNA molecules that have recently emerged as regulators of impaired wound healing and could be a target for DFU treatment. miRNA therapeutics can be delivered to the wound region using different delivery systems such as exosomes and nanoparticles. These wound dressings have a high potential for treating diabetic wounds by topical delivery of certain miRNAs in a sustained release manner.

## Introduction

Wound healing is a physiological process involved in restoring the integrity of tissue following an injury (1). The wound repair process involves coordinating four overlapping phases, including hemostasis, inflammation, proliferation, and remodeling (2). Hemostasis is the first healing step involving platelet activation and fibrin clot formation at the wound site. The inflammatory phase is initiated thereafter by releasing different growth factors, cytokines, and interleukins. Inflammatory cells like neutrophils are immediately recruited to the wound and produce antimicrobial proteases, pro-inflammatory cytokines, and other growth factors. Macrophages will also remove bacteria, foreign particles, and dead cells (3). Cells like dermal fibroblasts start proliferating through the proliferation phase to regenerate wounded tissues. These cells produce extracellular matrix proteins (ECM) such as collagen, hyaluronic acid, and fibronectin, which help generate a vascularized ECM, called granulation tissue (GT), to close the wound. The last phase of healing, remodeling, is associated with restructuring the ECM, collagen reorganizing, and scar formation (4). Chronic wounds are characterized by prolonged inflammation, infection, and tissue necrosis (5). Diabetic patients frequently suffer from developing chronic wounds. Approximately 15–25% of diabetic patients will be affected by diabetic foot ulcers (DFU) and may undergo amputation during disease progression (6). Various factors impair the healing process in diabetic wounds, including immune system deficiency, cellular dysfunction, low blood circulation, and deregulation of growth factor activity (7). Therefore, the management of DFU is a main therapeutic challenge and needs to find effective methods to promote the healing process (8). 

MicroRNAs (miRNAs) are a class of endogenous non-coding RNAs that have recently emerged as the key regulators of the wound healing process (9). Several miRNAs have been identified in the regulation of different stages of wound healing, and their impaired expression may contribute to the development or treatment of chronic wounds, especially diabetic ulcers (10). For example, miR-155 is expressed during the inflammatory phase and promotes cytokine production and growth factors. Inhibition of miR-155 can reduce inflammatory responses, decrease the accumulation of immune cells at the wound site, and improve the healing process (11). In contrast, miR-132-3p is up-regulated in the inflammatory phase and inhibits the expression of cytokines. MiR-132 expression is down-regulated in human diabetic ulcers and skin wounds of leptin receptor-deficient (db/db) diabetic mice compared with normal skin and wild mice, respectively. Topical administration of a mixture of functional miR-132 liposomes and pluronic F-127 gel mediated the anti-inflammatory and proliferative role in human *ex vivo* wounds (12). MiR-21 is a well-known miRNA that exhibits an anti-inflammatory effect by promoting cytokine and growth factor production. MiR-21 is also involved in the proliferative stage, and its overexpression increased keratinocyte migration while its knockdown impaired migration and re-epithelialization (13). MiR-146a has been reported to be down-regulated in diabetic mouse wounds and delayed the process of chronic wound healing. MiR-146a regulates the inflammation process by reducing pro-inflammatory target gene expression and therefore improves wound repair (10). The level of miR-31 is remarkably induced in keratinocytes at the wound edge in the inflammation and proliferation process and promotes re-epithelialization and wound closure (14). Another miRNA, miR-126, was increased in diabetic patients with DFU and played an essential role in wound healing by promoting angiogenesis and vascular network development. (10).

Standard wound treatment involves surgical debridement of necrotic tissues, antibiotic therapy, and using wound dressing to protect the wound from infection and speed up the healing process. Wound dressing materials can be classified into different groups: traditional, interactive materials, tissue-engineered skin substitutes, and bioactive dressings. Bioactive dressings, like hydrogels, hydrocolloids, collagen, and nanofibers, are novel forms of dressing materials. Hydrogel-based wound dressings are absorptive materials that can provide a moist environment at the wound site. Wound dressings composed of nanofibers have the advantage of promoting regeneration and accelerating healing. Moreover, bioactive dressings can deliver therapeutic agents such as antibiotics, growth factors, vitamins, or nanoparticles (7). 

**Figure 1:**
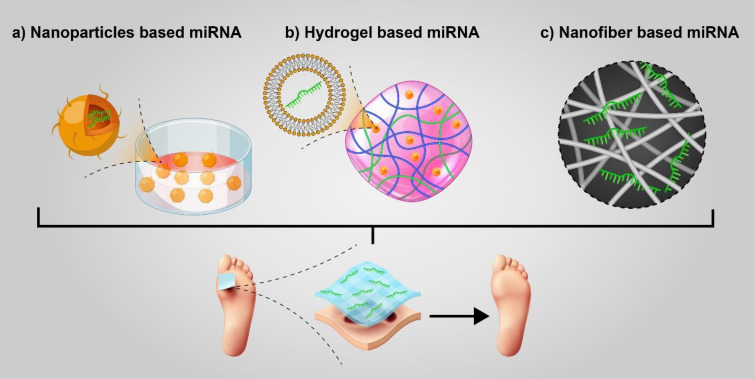
Schematic representations of miRNAs conjugated with bioactive dressings, including nanoparticles (a), hydrogels (b), and nanofibers (c). Integration of miRNAs into bioactive materials can accelerate the healing of diabetic wounds

The most recent developments in diabetic wound healing have focused on integrating miRNAs into the wound dressing (Figure 1). Wound dressings based on bioactive factors loaded with nanoparticles like miRNA-tagged nanoparticles would be a potential approach for accelerating diabetic wound healing. Sener *et al*. prepared a self-healable, flexible injectable, and anti-adhesive zwitterionic cryogel loaded with miR-146a conjugated cerium oxide (CNP) nanoparticles to promote wound healing in a homozygous diabetic mouse model. These non-toxic nanoparticles can reduce reactive oxygen species. Sustained release of miR-146a over time as an anti-inflammatory miRNA led to reduced IL-6 and CXCL2 and increased type 1 collagen. Based on their results, the wound perfectly healed six days earlier in the CNP-miR-146a conjugate treated group compared with the control zwitterionic gel treated group, following topical application of miRNA-loaded gel (15). In a recent study by Mulholland *et al*. electrospun polyvinyl alcohol (PVA) nanofiber patches were used to deliver plasmid encoding miR-31 electrostatic complexed with the CHAT peptide into the wound. Transfection of CHAT/pmiR-31 nanoparticles in human dermal microvascular endothelial cells (HMEC-1) and keratinocyte (HACAT) cells caused elevated cell migration; moreover, tubulogenesis and branch point formation were boosted significantly in HMEC-1 48 hr post-transfection compared with untreated cells. Besides *in vitro* investigations, they evaluated CHAT/pmiR-31 loaded nanofibers in full-thickness wounding model surgery. In addition, the angiogenesis activity and epidermal thickness increased in the groups treated with CHAT/pmiR-31 loaded nanofibers compared with the untreated groups (16). In another study, it has been shown that chitosan hydrogel loaded with exosomes secreted from miR-126-3p overexpressing synovium mesenchymal stem cells (SMSCs) noticeably promoted wound closure through increased re-epithelialization, granulation, and angiogenesis in a diabetic rat model at day 14 compared with untreated cells or those treated with chitosan alone (17). Collagen hydrogel containing human adipose tissue-derived mesenchymal stem cells (ADSCs) secretome demonstrated high angiogenic potential in human skin endothelial cells compared with unloaded hydrogel, which was partially associated with high expression levels of secretome-derived pro-angiogenic miRNAs such as miR-126, miR-296, miR-210, and miR-378 (18); however, *in vivo* or *ex vivo* wound model evaluation of the results were not done. Some biomaterials could regulate miRNA expression. *In vitro* and *in vivo* experiments revealed that graphene oxide-derived biomaterial (hydrogel) could promote infectious wound healing by up-regulating miR-21/PI3K/Akt and down-regulating IL-17 in ADSCs-derived exosomes (19). 

## Conclusion

Altogether, due to challenges in chronic wound repair, identification of novel approaches for accelerating wound closure is inevitable. Sustained release of miRNAs by using ideal wound dressings like chitosan hydrogel or nanofiber loaded with therapeutics like miRNA-modified exosomes may be a potential exciting method in diabetic chronic wound recovery. The current research on miRNAs in wound dressing is still in the early stages. Hence, preclinical and clinical investigations are necessary to confirm miRNA-based therapeutic applications. 

## Authors’ Contributions

MHS Provided the concept; MHS and NM Wrote and edited the manuscript. Both authors have read and approved the manuscript.

## Conflicts of Interest

No conflicts of interest were declared.
